# Opium Antidote

**Published:** 1877-03

**Authors:** 


The following is taken from *Ohio Medical Recorder* of February, 1877:

The opium antidote business has been sharply called upon to halt! by the Cumberland Medical Society of Maine. They have caused a quantitative analysis of certain of these nostrums to be made, and report the results widely among the profession. One specimen, manufactured by Mrs. J. A. Dollinger, of La Porte, Indiana, was analyzed by Walz and Stillwell, New York city, who found it to consist of glycerine colored with aniline red, and to contain in solution 1.383 per cent. by weight of the sulphate of morphia—about seven grains to the ounce. The second was the preparation of "Dr. S. B. Collins, the great Narcologist of the Age," also of La Porte. The analysis of this was made by Dr. Henry Carmichael, Assayor of the State of Maine, and differed from the preceding only in the amount of the sulphate of morphia shown to be present, namely, 3.2 per cent. A teaspoonful (a dose frequently prescribed by the proprietor) would contain almost two grains of the morphia—nearly twelve times the ordinary medicinal dose.

